# Emerging Infectious Literatures and the Zombie Condition

**DOI:** 10.3201/eid2409.170658

**Published:** 2018-09

**Authors:** Joanna Verran, Xavier Aldana Reyes

**Affiliations:** Manchester Metropolitan University, Manchester, UK

**Keywords:** zombies, fiction, epidemiology, Manchester, United Kingdom, bioterrorism and preparedness, prions and related diseases

## Abstract

The book club format has enabled expert and nonexpert exploration of infection and epidemiology as encountered in popular literature. This exploration reveals that fiction focusing on apocalyptic disease often uses the zombie as embodiment of infection, as well as an exemplar of current knowledge on emerging disease.

The Bad Bugs Book Club (https://www2.mmu.ac.uk/engage/what-we-do/bad-bugs-bookclub/) was established in 2009 (*1*). This reading group meets every 2 months to discuss works of literary fiction from any genre that features infectious disease. The aim of these meetings is to engage scientists and nonscientists in discussions about epidemiology and infection and to consider what the texts tell us about our perception of science and its advances. 

Book clubs, or reading groups, have increased in popularity since the late 1990s. Estimates in 2003 were of ≈50,000 book clubs in Britain and ≈500,000 in the United States (*2*). Some clubs are specialized groups whose members read restricted genres such as crime fiction, science fiction, or the classics. Fiction and nonfiction texts focusing on microbiology have been incorporated into the book club format, led primarily by academics for student education (*3**–**5*), but no evidence has been found in the literature for such groups for the general public. 

Adults have been identified as 1 of 3 key underserved audiences in terms of engagement with science (*6*). The reading group format addresses this need and contrasts with unidirectional science communication activities from scientists (experts) to members of the public (nonexperts) (*7*) in that reading groups provide an opportunity for adults to contribute their knowledge, experience, and perceptions about the reading subject matter on a level platform.

Bad Bugs Book Club meetings take place in an informal environment (a bar) in the evening, typically comprising up to 8 participants, of whom around half have been members since 2009. New members are welcome; meetings are advertised online, as well as through an email group. At each meeting one book is discussed, selected by the group at the previous meeting. Discussions tend to be led by the group leader (J.V.), but all members can lead discussions, particularly for books that they have suggested. The meeting leader prepares questions before the meeting to guide discussion and publishes them online on the book club’s website after the meeting, but usually conversation does not require prompting. Meeting reports are also posted online, enabling themes to be identified across books and genres, as well as establishing a rich, freely accessible resource that has informed much of the content of this article. 

Our findings, based on the reports accessible from the book club’s website, show that fiction content in epidemiologic narratives is often influenced by epidemiologic outbreaks—authors absorbing and recasting what have been called “outbreak narratives” (*8*) within plotlines—as well as by the pervading rhetoric of fear that surrounds pandemics in the media (*9*). We found that the representation of vampires and, particularly, zombies as agents of infection was frequent; these monsters appeared often as epidemiologic avatars (*10*–*12*). This article therefore examines the role of the zombie as a metaphor for infectious disease and the emergence of new literature describing apocalyptic disease as examples of the ways in which fiction can lead to a widespread discussion and understanding of pandemics. We use examples from books discussed in our book club meetings.

The Zombie Research Society defines a zombie as “a relentlessly aggressive human or reanimated human corpse driven by a biologic infection” (http://zombieresearchsociety.com/about-us). This description neatly summarizes the current state of zombies, both narratively (in the stories told about them) and in terms of how they might become useful in our understanding of pandemics, contagion patterns, and prevention. One aim of the book club is to redress the balance between fear of infection and the importance of a working knowledge of microbiology, but zombies also provide a useful means for examining concerns about fast-spreading diseases in the first world, the “shock doctrine” used in the reporting of pandemics (*13*), emerging disease, and, more recently, the impact of antimicrobial resistance. Indeed, Bishop (*14*) proposes that “post-9/11 anxieties about potential terrorist attacks via anthrax, avian influenza, swine flu, and other forms of biologic warfare” may be responsible for this emergence and suggests that apocalyptic contagion narratives might outlive interest in the zombie.

## The Zombie as Allegory of Infectious Disease Epidemiology

In fiction focusing on infectious disease, the invisible pathogen is an embodiment of the unknown, existing in intimate contact with us, yet beyond the boundaries of our senses. The infection is carried by its host and transmitted to another; its effects become apparent as symptoms develop. The pathogen as a microscopic Gothic presence can be represented metaphorically and macroscopically in the figure of the zombie, much as the ghost, the undead (the vampire), and the “weird creature” have traditionally acted as springboards for the exploration of the beyond and the numinous (*15*–*17*). In the zombie, internal damage to the host becomes externalized, and contagion patterns among populations are demonstrated as the zombie hordes rampage. With no subclinical manifestation, the zombie makes the apocalypse visible, enabling us to physically map the spread of infection. In other words, the zombie becomes an “allegory of infectious disease” and a “metaphor of ubiquitous contagion” (*18*). In their hordelike structure, zombies also operate metonymically, standing in for large swaths of the population (the infected), or viruses (the infection). The mathematics of zombie outbreaks has therefore also been explored as an education tool to represent contagion patterns and containment strategies (*19*,*20*).

In 1996, the influential horror survival video game *Resident Evil* was developed by Capcom (Capcom USA, San Francisco, CA, USA); this release was the first zombie game to rely on infection as the catalyst for the zombie state. Since then, and particularly after 2002, when a spate of infection zombie texts emerged (such as the *Resident Evil* movie series [2002 onward; directed by Paul W.S. Anderson], *28 Days Later* [directed by Danny Boyle, 2002], and *The Walking Dead* comic series [2003–present] [*21*]), infection has tended to become the primary cause of the zombie condition itself. A blurring of the lines between the traditional zombie and the rabid human has shortened the distance between fantasy and reality (*22*). The zombie dominates the horror fiction landscape because it has adapted well to the real-life scenario of pandemic outbreaks as represented by and in the media. Like microorganisms themselves, zombies respond well to selection pressures.

Other parallels between the infection process and the infectious zombie text are apparent. In 2007’s *28 Weeks Later* (directed by Juan Carlos Fresnadillo), a woman and son appear to be immune to infection but carry the Rage virus to susceptible populations. The novel *I Am Legend* (*10*) describes the scientific methods applied to isolate the cause of the undead plague, but ultimately it is the evolution of the agent of zombie infection that enables the survival of the host and the pathogen in the novel (although the monsters are infected with the “vampiris bacillus,” their behavior by night is zombie-like). Likewise, in *The Girl with All the Gifts* (*23*), airborne fungal spores ultimately bring about the extinction of the human race: the new world is populated by partially immune, but infected, children. Killing the host limits spread of infection and survival of the pathogen. In these novels, symbiosis is advantageous to both partners in the host–parasite relationship.

Tolerance of infection leads to recovery in the novel *Warm Bodies* (*24*); the immune response is stimulated when the host begins to interact socially with humans. *Breathers: A Zombie’s Lament* (*25*) is narrated by a zombie who regains his self-confidence through attendance at “Undead Anonymous” meetings and becomes a champion for zombie rights (with a taste for human flesh). In comparable texts in other media, such as the television series *In the Flesh* (written and produced by Dominic Mitchell, 2013–2014) and *iZombie* (directed by Rob Thomas, 2016–present), zombies are also likable main characters who suffer at the hands of a society that does not understand them. This cross-media development suggests that the zombie condition is evolving heterogeneously, sometimes (especially in melodrama and romance fiction) moving away from the image of the monstrous apocalyptic vector and into a more individually focused host–parasite relationship. The sentient zombie of *Breathers* or *Warm Bodies* can cohabit the same cultural space as the more traditional aggressors of Seth Grahame-Smith’s book *Pride and Prejudice and Zombies* (*26*) and Darren Shan’s *Zom-B* (*27*) series, and even zombie-like creatures, such as the rabid attackers of David Moody’s *Hater* (*28*). What unites all these zombies is a similar approach to the cause of their ontological status, namely, infection as the point of origin.

In contemporary zombie fiction, 3 different contagion outcomes predominate that parallel the pathogenesis of infection: success of the predator, mutualism, or a defeat of the predator. Our innate knowledge of real disease epidemiology is thus illustrated in much zombie literature by the behavior of the humans who are under threat. In the absence of any treatment strategy, options are restricted to quarantine (isolation of the infected, as in Cherie Priest’s *Boneshaker* [*29*]), immunization strategies (protection of the uninfected in *Warm Bodies*, Charlie Higson’s *The Enemy* [*30*], and Jonathan Maberry’s *Rot and Ruin* [*31*]), and control (extermination of the agent in Max Brooks’ *World War Z* [*32*]). As zombies become the manifestation of virulent infection, they do not just address our fear of pandemic disease and apocalypse; they also allow us to explore coping strategies.

## Pathogens Influencing Zombie Epidemiology

Viruses are the perfect mechanistic microbiological comparison for the zombie, whose sole function is essentially to replicate/transmit the infection. Although some bacterial infections have had an impact on a global scale, viral pandemics are a greater threat: viruses replicate inefficiently, frequently creating different versions of themselves against which we have reduced or no immunological defense (*33*). The attributes of airborne transmission, high infection rate, and high virulence are the worst possible outcome for humanity because airborne transmission is extremely difficult to control or prevent, a high infection rate ensures high numbers of cases, and high virulence results in high rates of illness and death (as depicted in the 2011 infectious disease–themed movie *Contagion,* directed by Steven Soderbergh). In addition, the incubation period needs to be sufficiently long to enable others to become infected. These patterns have been followed in popular fictional narratives. For example, Ebola virus disease has varying infectivity and virulence: in the 1976 Zaire and Sudan outbreaks, infectivity was relatively low (contact with infected fluids was the route of infection), but fatality rates were high, approaching 90%, whereas in the 2014 outbreak, the fatality rate was 50% (*34*). Ebola epidemiology enabled dramatic scenarios in the book *The Hot Zone* (*35*) and the movie *Outbreak* (directed by Wolfgang Petersen, 1995). The zombie apocalypse draws from such scenarios, yet simultaneously eclipses them all in its scale.

The epidemiology of viral infection we have described may not always be directly applicable to the spread of the zombie condition (e.g., airborne infection happens only when the zombie origin is fungal), but noteworthy patterns do emerge. The novel *World War Z* (as opposed to the 2013 movie of the same name, directed by Marc Forster, which bears little resemblance to the novel) is a good example of how zombie fiction uses real epidemiologic scares to shape the ultimate viral zombie horror narrative. In microbiological terms, the book describes the emergence and spread of a pandemic whose infection and mortality rates are 100%, with an incubation period of a few days, whose symptoms make those infected extremely dangerous to society, and for which there is no treatment. Inactivation of infected persons by destruction of the brain becomes the only solution and prevention strategy. The infection is not airborne; rather, it is transmitted by biting or entry of infected tissue through injured skin and via transplants. Still, its other traits correlate with those of several true infectious agents, such as rabies virus, Creutzfeldt-Jakob prion disease, cytomegalovirus, herpes virus, and HIV (*33*). The zombie incubation period in the novel is also extended (“slow burns”) if a major blood vessel is missed during biting. This particular aspect of the virus is itself borrowed from rabies, in which a longer incubation period results from a bite to the leg rather than a bite to the neck. As with many influenza pandemics and severe acute respiratory syndrome, *World War Z*’s pandemic begins in China. In this novel, there are other localized outbreaks, but the pandemic develops via misinformation and obfuscation—as occurred with the spread of severe acute respiratory syndrome from China. Politics plays a major part in the spread of the pandemic.

The zombie, an insentient creature with a tendency to swarm, has been used in several disciplines in recent years to shed light on the dynamics of economics, capitalism, and international politics and to channel fears connected to social alienation, especially as a result of digital and communication technologies and overpopulation (*22*,*36*–*38*). In contrast to the intellectual and allegorical use of the zombie in such disciplines, articles describing the epidemiologic properties and preventive measures in the event of a “zombie outbreak” have been presented in the scientific literature in a more ironic tone, nevertheless taking cues from emerging public interest in zombies. For example, *BMJ* has provided information on epidemiology, treatment, and prevention (*39*). However, the use of zombie epidemiology as an education tool requires careful planning; for instance, the CDC’s section on “zombie preparedness” (http://www.cdc.gov/phpr/zombies.htm) has been accused of “trivialization” of the preparedness topic (*40*).

At least 1 of the popular reimaginings of the late postmillennial zombie proposes that zombified humans may have a “new strain of prion disease.” In the novel *Zombie Autopsies: Secret Notes from the Apocalypse* (*41*), the private notes of a neurodevelopmental biologist written in a remote laboratory setting “where the world community could focus its efforts on the scientific study of ANSD [Ataxic Neurodegenerative Satiety Deficiency Syndrome]” describe the dissections of 3 zombie subjects before the author succumbs to the disease himself. This narrative is framed as the main section of a highly confidential memorandum from the United Nations outpost. Two working hypotheses on the nature of the pandemic are proposed. The first is that ANSD may be caused by an airborne engineered plague, a symbiosis that would result in 3 contagions operating through a single vector, specifically a combination of influenza and prion and a third unknown infectious agent. The second option is that humans may be faced “with something new… with distinct and adaptive properties. Something that hijacks the host.” 

Two different fictional worlds, in Charlie Higson’s *The Enemy* series (2009–2015) (*30*) and *The Girl with All the Gifts* (*23*), adopt and modify the “zombie fungus” *Ophiocordyceps unilateralis* as the apocalyptic zombie agent. In both cases, airborne fungal spores provide an inescapable source of infection, with the sporulation cycle being critical to the plot. Mira Grant’s novel *Feed* (*42*) is the first volume in a series following life in a postapocalyptic America where a third of the population has succumbed to the Kellis-Amberlee virus; zombies are a result of an ecoterrorism act that “released a half-tested ‘cure for the common cold’ into the atmosphere.” The novel’s dormant pathogen is based on *Toxoplasma gondii*, a pathogen that would not wipe out the entire susceptible population. In all these new novels, the aim is to provide a microbiologically accurate backdrop and story in which the population either succumbs to infection or learns to cope with a pathogen—for example, in *Feed*, through out-of-bounds contamination areas, complex decontamination routines, and constant screening.

Books like *Zombie Autopsies* shows how new infectious zombie texts act as virological repositories. The opposite is also true; since *I Am Legend*, several publications have attempted to use the symptoms of zombieism to explain the workings of specific pathogens and scientific principles. Many examples exist; a particularly notable one is *Do Zombies Dream of Undead Sheep? A Neuroscientific View of the Zombie Brain* (*43*), largely an introduction to neurology, in which consciousness deficit hypoactivity disorder (CDHD) is deemed to be the result of infection from external pathogens that hijack human systems, which could be caused by either *Cordyceps*-style fungi (as in *The Girl with All the Gifts*), prion disease, or evolved brain tapeworms (parasites or protozoans). The zombie status is used here to explain viral attacks on the brain, as well as to describe how this organ generally operates. A book like *Do Zombies Dream of Undead Sheep?* shows both the tremendous reach of the zombie in the 21st century and how fiction may, in turn, end up delivering the very science it uses as inspiration.

## Conclusion: Zombies and Emerging Infectious Literatures

New infectious zombie texts evince the main shifts in fictional representations of infection narratives; in them, symptoms and epidemiology are often based on real infections. We have termed the wider phenomenon within which the zombie narrative has manifested “emerging infectious literatures,” an echo of the term “emerging infectious diseases.” Generically, emerging infectious literatures are varied: some show clear horrific leanings, whereas others are more obviously defined as science fiction or thrillers. Influenza is a particularly malleable candidate for such narratives; the varying possible rates of transmission and virulence have been used to frame different postapocalyptic scenarios since the publication of Stephen King’s *The Stand* (*44*). For example, in *Immunity* (*45*), 4% of the population is lost in a matter of months, and screening is deployed to detect the infected, coupled with immunization of selected persons. In *Station Eleven* (*46*), a much more virulent strain almost wipes out humanity; survivors are few and required to construct new, small, civilizations. Yet another book, *Not Forgetting the Whale* (*47*), focuses on how an isolated Cornish community manages to avoid succumbing to an outbreak affecting the urban environment. Novels about the future impact of antimicrobial resistance are as yet few and far between, but no less interesting: *A Fierce Radiance* (*48*) describes the industrial production of antimicrobial drugs during World War II, the prioritization of combat troops to receive treatment, and the impact of antimicrobial drugs on public health, and *The Deep Zone* (*49*) is concerned with the discovery of new antimicrobial drugs in unusual environments (caves), couched in industrial and political espionage. More recently, short stories (for example, *Infectious Futures*, published by NESTA [http://www.nesta.org.uk/search?search_api_views_fulltext=Infectious%20futures]), comic books (*50*), and other public information efforts are attempting to raise awareness and change behavior. Perhaps Zika virus and Middle East respiratory syndrome will provide inspiration for the next epidemiologic antiheroes. Zombies will likely remain a returning concern for epidemiologically inclined writers.

To return to our initial premise, the book club format successfully allows discussion between experts and nonexperts about the overlaps between pandemic fact and fiction. Through these discussions, participants can focus on key messages about disease and infection that underpin the fiction narrative. Meeting reports and reading guides posted on the Bad Bugs Book Club website over the past 9 years provide evidence of the success of multiway discussion, and are a rich resource for others wishing to engage in similar activities. Book club discussions have enabled identification of different themes emerging from such texts, the most notable of which has, for us, been the zombie infection narrative. In the case of the novels discussed throughout this article, our meetings helped us come to grips with the contemporary significance, porosity, and ubiquity of the zombie as contemporary monster. The zombie has enabled the exploration of our behaviors when confronted with infection and served as an indication of how fiction reflects current knowledge about pandemics. In the same way that the changing virulence of pathogens has occurred throughout history, the zombie trope is flexible, something that has enabled its survival in 21st century literature. Emerging infectious diseases and their management likewise provide a rich lode for continuous exploration of the microbiological present and potential future in fiction.
